# Synergistic Effects of Rotenone and Abamectin on Physiological Suppression, Population Inhibition, and Ion Disruption of *Bursaphelenchus xylophilus*

**DOI:** 10.3390/ijms26189133

**Published:** 2025-09-18

**Authors:** Quanhong Zhang, Lili Hu, Liusheng Chen, Yongliu Jiang, Danyang Zhao, Gaofeng Cui

**Affiliations:** 1 Guangdong Provincial Key Laboratory of Silviculture, Protection and Utilization, Guangdong Academy of Forestry, Guangzhou 510520, China; hqz5321@163.com (Q.Z.); hulili0113@163.com (L.H.); lshenchen2008@163.com (L.C.); jiangyliu@163.com (Y.J.); 2 College of Plant Protection, South China Agricultural University, Guangzhou 510642, China

**Keywords:** *Bursaphelenchus xylophilus*, rotenone, abamectin, synergistic effects, physiological activity, population dynamics, ion disorder

## Abstract

Pine wilt disease, which is induced by pine wood nematode (PWN, *Bursaphelenchus xylophilus*), has caused huge economic and ecological losses. To overcome the drawbacks of chemical control against PWN, twenty compounds were screened, and a synergistic botanical–chemical combination was identified. A proportion of abamectin to rotenone of 7:3 (5.73 and 1.78 mg/L, respectively) achieved the highest co-toxicity coefficient of 231.09 with a median lethal concentration of 3.18 mg/L. It revealed 0% mortality in *Pinus massoniana* seedlings at 60 days post-treatment when applied at 400 times the synergistic concentration (2.29 g/L abamectin + 0.71 g/L rotenone) at 7 days after PWN inoculation. Furthermore, the synergistic combination significantly affected the physiological activity and population dynamics of PWN. Female oviposition was reduced by 71.92%, the egg hatching rates declined to 13.09 ± 0.02%, and head thrashing frequency was inhibited by 99.23 ± 0.01%. The enzymatic activities of peroxidase, acetylcholinesterase, succinate dehydrogenase, and glutathione S-transferase were significantly increased, while the population size declined by 96.17%. Transcriptomic and gene expression analyses suggested a potential “Na^+^/Ca^2+^/Cl^−^ ionic storm,” since the synergistic combination significantly activated genes associated with voltage-gated calcium channels, glutamate-gated chloride channels, and amiloride-sensitive sodium channels. These findings provide an eco-friendly strategy for PWN management via chemical control.

## 1. Introduction

Pine wilt disease (PWD) is a globally significant quarantine forest disease caused by pine wood nematode (PWN), *Bursaphelenchus xylophilus*. It causes destructive damage to pines and leads to huge economic and ecological losses [1,2,3,4]. PWN is native to North America and has spread to many countries in Asia and Europe through commercial transportation. The host range of PWN includes 47 *Pinus* and 14 non-*Pinus* tree species, with the main insect vectors being *Cerambycidae* species [5,6]. PWN is thought to disrupt the resin canals and vascular tissues of pine trees, thereby impairing water transport, and ultimately leading to rapid wilting and death [7,8,9]. Currently, the main strategies for PWD management include disease quarantine, epidemiological surveillance, removal of infected trees, control of vector beetles, and the injection of nematicides into pine trees [1,2,3,4,10,11]. Chemical pesticides play vital roles in controlling insect vectors and nematodes due to their rapid and effective action, broad-spectrum nematicidal activity, and strong controllability [12,13,14]. For example, abamectin and emamectin benzoate are the main insecticidal compounds applied via trunk injection in East Asia. They target glutamate-gated and γ-aminobutyric acid (GABA)-gated chloride channels, disrupting neural signal transduction [15,16,17]. However, large-scale pesticide application using drones and long-term nematicide injection can lead to resistance development and have adverse environmental effects [18,19,20]. Therefore, more efficient, environmentally-friendly, and sustainable pesticides are needed for PWD control.

Developing new insecticidal ingredients and screening pesticide combinations with synergistic effects are key priorities in pest management. For instance, botanical compounds from 749 plant species across 171 families have been screened for anti-PWN activity since 1980, and a total of 555 bioactive compounds and 433 synthetic derivatives have been identified as exhibiting nematicidal effects [21]. Rotenone, a specific inhibitor of mitochondrial complex I, has demonstrated exceptional efficacy against PWN, with a median lethal concentration (LC_50_) of 1.86 μg/mL [22]. However, only a few plant- derived compounds, such as rotenone and matrine, have been applied in practice. Pesticide combination has become another important approach to enhance efficacy and delay resistance. Studies have shown that abamectin-fluensulfone combinations reduce the LC_50_/24 h for PWN to 2.4 mg/L, outperforming other combinations [23]. The abamectin-fluopyram combination showed optimal laboratory performance against PWN with an LC_50_ of 2.0208 mg/L and an egg hatching inhibition rate of 20.32% [24]. Moreover, multi-target mechanisms and reduced dosage can further minimize environmental impact [12,14,25]. Nonetheless, current research mainly focuses on optimizing combinations of synthetic pesticides, which may still involve high total chemical loads and environmental risks. Thus, integrating synthetic pesticides with botanical active ingredients has emerged as a critical strategy to overcome these limitations. Synergistic formulations could minimize reliance on synthetic pesticides without compromising efficacy or stability, offering cost-effective and scalable solutions for integrated pest management.

In this study, twenty chemical and biogenic active ingredients were screened, and an abamectin-rotenone combination with a synergistic effect was developed. Indoor pot trials were conducted to validate the control efficacy of the synergistic combination. Furthermore, population dynamics, physiological impacts, and genetic changes were investigated to elucidate the underlying synergistic mechanism. These results provide a practical foundation for chemical control strategies and offer a green, efficient solution for PWD management.

## 2. Results

### 2.1. Nematicidal Activity of Chemical and Biogenic Active Ingredients Against PWN

The toxicity of 20 chemical and biogenic active ingredients against PWN was evaluated using the immersion method. As shown in Table 1, fluopyram, a chemical compound, exhibited the highest nematicidal activity with an LC_50_ of 2.25 mg/L at 24 h, followed by emamectin benzoate (LC_50_ = 5.98 mg/L) and abamectin (LC_50_ = 8.18 mg/L). Fosthiazate, fluensulfone, and thiamethoxam demonstrated poor efficacy. Among botanical ingredients, rotenone showed the highest activity (LC_50_ = 5.94 mg/L), which was comparable to that of chemical pesticides. Harmine, curcumin, spermidine, and ethyl allicin also showed potent nematicidal activity, with LC_50_ values of 68.07, 80.05, 87.82, and 91.08 mg/L, respectively. Azadirachtin, matrine, and camptothecin exhibited low toxicity to PWN.

### 2.2. Interaction Assay for Compound Combinations Against PWN

The synergistic effects of emamectin benzoate, abamectin, and fluopyram in combination with rotenone were systematically evaluated using the interaction assay. As shown in Table 2, the abamectin-rotenone combination displayed the strongest synergy. With the exception of groups 1, 2, and 11, all other ratios of abamectin-rotenone combination exhibited synergistic effects, with toxicity ratios of 1.69, 1.68, and 1.62 at ratios of 7:3, 6:4, and 5:5, respectively. For the emamectin benzoate-rotenone combination, groups 3, 4, 5, 6, and 10 showed synergistic effects, with toxicity ratios ranging from 1.34 to 1.52. The highest mortality was observed at the 7:3 ratio. However, the combination of fluopyram and rotenone exhibited merely additive effects across all ratios, with the toxicity ratios ranging from 0.75 to 1.25.

### 2.3. The Co-Toxicity Coefficient (CTC) Method for Compound Combinations Against PWN

The top three ratios of the emamectin benzoate-rotenone and abamectin-rotenone combination were verified using the CTC method. As shown in Table 3, the emamectin benzoate-rotenone combination exhibited optimal nematicidal activity at the 7:3 ratio, with an LC_50_ of 4.00 mg/L. This value was significantly lower than the LC_50_ values for the 6:4 (4.11 mg/L) and 5:5 (4.32 mg/L) ratios. Similarly, the abamectin-rotenone combination demonstrated strong synergistic effects, achieving the highest CTC value of 231.09 at the 7:3 ratio. It also resulted in the lowest LC_50_ of 3.18 mg/L, while the LC_50_ values for the 6:4 and 5:5 ratios were 3.61 mg/L and 3.76 mg/L, respectively.

The optimal synergistic combination, abamectin-rotenone at a 7:3 ratio (at concentrations of 5.73 mg/L abamectin and 1.78 mg/L rotenone), was selected for practical application tests and subsequent studies on its synergistic effects and mechanisms. Accordingly, four treatment groups were established, including abamectin (5.73 mg/L, SCA), rotenone (1.78 mg/L, SCY), the synergistic combination (abamectin 5.73 mg/L + rotenone 1.78 mg/L, SCF) and a control group (dimethyl sulfoxide, DMSO, ≤1%, CK).

### 2.4. Indoor Pot Trials of Synergistic Combination Against PWN

**Figure 1:**
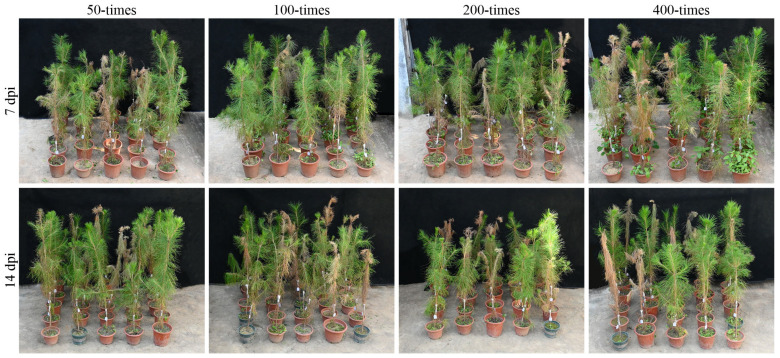
Control effects of different reagents on the PWN-inoculated *P. massoniana* seedlings (60 d). The order of treatments in each photo is water control, DMSO control, rotenone (SCY), abamectin (SCA), and the abamectin-rotenone combination (SCF) from left to right.

Typical symptoms of PWD, including wilting apical needles and yellowing leaves, were observed at 14 days post-inoculation (dpi) with PWN on 3-year-old *Pinus massoniana* seedlings, with complete death occurring at approximately 30 dpi. Reagent solutions at concentrations 50-, 100-, 200-, and 400-times the laboratory concentration were sprayed on the seedlings at both 7 and 14 dpi. This corresponded to abamectin concentrations ranging from 286.50 to 2292.00 mg/L and rotenone concentrations from 89.00 to 712.00 mg/L, respectively. A 60-day observation period followed (Figure 1).

The lower-concentration treatments (50-, 100-, and 200-times) of SCY, SCA, and SCF demonstrated limited disease control efficacy at both 7 and 14 dpi, showing no significant reduction in seedling mortality compared to the control group. Notably, the application of the 400-times SCF treatment at 7 dpi completely prevented PWD, resulting in 0% seedling mortality at 60 dpi. In contrast, other treatments at 7 dpi resulted in mortality rates of 20% for SCA, 40% for SCY, 60% for the DMSO control, and 80% for the water control. When applied at 14 dpi, the 400-times SCF treatment resulted in a mortality of 20% at 60 dpi, which was better than that of SCA (40%), SCY (60%, with two symptomatic plants), and the DMSO (80%) and water control (80%). A comparative analysis of application timing revealed that the early application (7 dpi) of the 400-times reagent treatments yielded lower mortality rates, indicating that preventive application is more effective than therapeutic treatment.

### 2.5. Morphological Observation of PWN After Treatments

To elucidate the underlying mechanism, morphological changes, physiological responses, population dynamics, and transcriptional differences were all analyzed after treatment with the synergistic combination.

**Figure 2:**
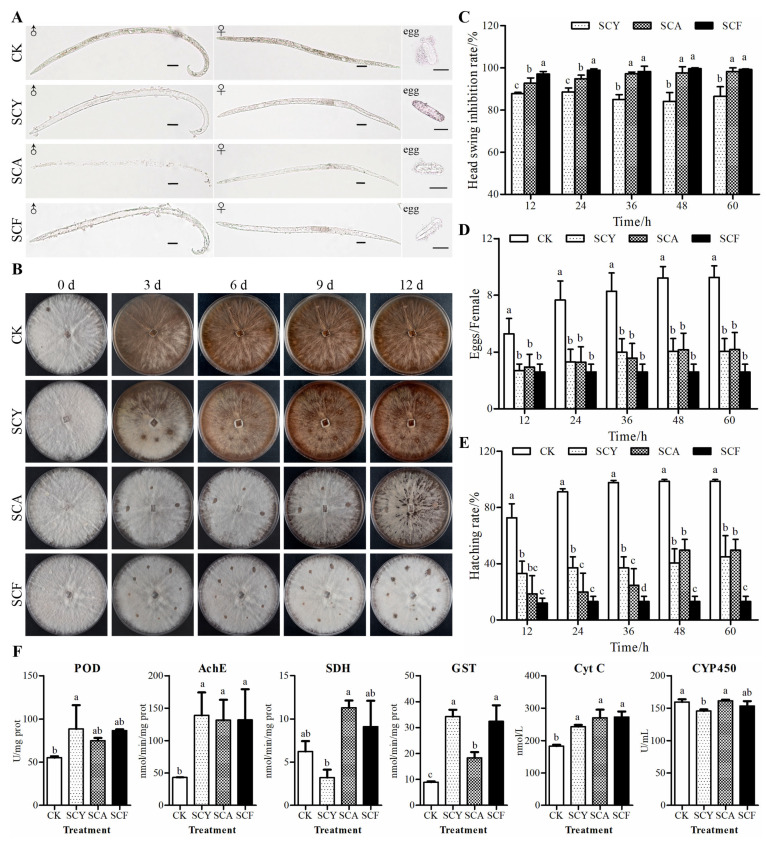
Effects of different treatments on the morphology and physiology of PWN. (**A**) Morphology. Scale bars: 20 μm. (**B**) Feeding behavior. (**C**) Head swing. (**D**) Oviposition. (**E**) Egg hatching rate. (**F**) Enzyme activities. POD: Peroxidase; AchE: Acetylcholinesterase; SDH: Succinate dehydrogenase; GST: Glutathione S-transferase; Cyt C: Cytochrome C; CYP450: Cytochrome P450. CK: DMSO; SCA: Abamectin (5.73 mg/L); SCY: Rotenone (1.78 mg/L); SCF: Abamectin + rotenone (5.73 mg/L + 1.78 mg/L). Different lowercase letters indicate significant differences between groups (*p* < 0.05).

Morphological changes of PWN were observed (Figure 2A). Living PWN typically exhibited S-shaped, coiled, wavy, or helical postures. After treatment, the nematodes initially exhibited transient paralysis accompanied by a marked attenuation of locomotory capacity. Mortality increased over time, accompanied by body stiffening (corpse rigidity) and morphological shifts to J-shaped or C-shaped forms. Subsequently, gas bubbles gradually emerged on the nematode cuticle, followed by progressive internal disintegration into fragmented tissue masses. However, there is no significant differences in these morphological changes were observed among the SCA, SCY, and SCF treatment groups.

### 2.6. Effects on Feeding Behavior of PWN

Continuous monitoring of PWN feeding behavior revealed significant differences among treatments (Figure 2B). Fungal plate assays demonstrated substantial feeding activity in the SCY and control groups at 3 d, which culminated in the complete consumption of surface mycelia within 6 days. In contrast, the SCA treatment showed no feeding traces until 9 days and achieved near-complete mycelia consumption by 12 days. Notably, no detectable feeding traces were observed in the SCF group throughout the experimental period.

### 2.7. Effects on Head Oscillation Behavior of PWN

All experimental treatments significantly inhibited the head oscillation behavior of PWN (Figure 2C). The SCA, SCY, and SCF groups consistently maintained inhibition rates exceeding 80% throughout all observational intervals. The SCF treatment exhibited a time-dependent enhancement, achieving an inhibition rate of 99.23% ± 0.01% at 60 h. A similar trend was observed in the SCA group, reaching 98.24% ± 0.01% inhibition at 60 h. In contrast, the SCY group showed slight fluctuations in inhibition, with the lowest inhibition rate of 84.15% ± 0.02% at 48 h.

### 2.8. Effects on Oviposition of PWN

All treatment groups exhibited significant inhibition of oviposition compared to the control (Figure 2D). However, there were no significant differences among the treatments. The SCF group demonstrated persistent inhibitory effects, maintaining egg production at 2.60 ± 0.25 eggs/female throughout the experimental period. In contrast, the SCA group showed a gradual attenuation of inhibitory efficacy over time, with the mean oviposition counts progressively increased from 2.94 ± 0.40 (12 h) to 4.18 ± 0.53 (60 h) eggs/female. The SCY group displayed a similar trend to that of the SCA group. Notably, the control group exhibited a significant increase in egg production over time, escalating from 5.28 ± 0.49 to 9.26 ± 0.37 eggs/female during the observation period.

### 2.9. Effects on Egg Hatching Rate of PWN

The egg hatching rates for the different treatments are presented in Figure 2E. The control group exhibited a typical hatching progression, achieving a peak rates of 98.68% ± 0.01% at 60 h post-treatment. The SCF group exhibited the strongest inhibitory effect, with a final hatching rate of 13.09% ± 0.02% at 60 h, which was significantly lower than that of all other groups. Moderate inhibition was observed in the SCA and SCY groups, with final hatching rates of 49.59% ± 0.03% and 44.89% ± 0.07% at 60 h, respectively.

### 2.10. Effects on Enzyme Activities of PWN

The enzyme activity profiles for different treatments are shown in Figure 2F. Peroxidase (POD) activity was lowest in the control group (55.19 ± 0.94 U/mg) and highest in the SCY group (88.55 ± 15.87 U/mg). Acetylcholinesterase (AchE) activity in the control group (43.07 ± 0.12 nmol/min/mg) was significantly lower than in the other treatments (SCY: 138.85 ± 20.46, SCA: 131.61 ± 18.11, and SCF: 131.88 ± 27.21 nmol/min/mg). However, no significant differences were detected among the treatment groups. Succinate dehydrogenase (SDH) activity was highest in the SCA group (11.30 ± 0.60 nmol/min/mg), followed by the SCF group (9.12 ± 2.12 nmol/min/mg) and the control group (6.23 ± 0.85 nmol/min/mg), with the SCY group showing the lowest activity (3.22 ± 0.65 nmol/min/mg). Glutathione S-transferase (GST) activity was significantly elevated in the SCY group (34.25 ± 1.51 nmol/min/mg), the SCF group (32.40 ± 4.40 nmol/min/mg), and the SCA group (18.32 ± 1.28 nmol/min/mg), compared to the control group (8.91 ± 0.24 nmol/min/mg). Cytochrome C (Cyt C) activity was also markedly enhanced in all treatment groups (SCF: 272.78 ± 9.79, SCA: 270.44 ± 14.56, SCY: 243.03 ± 3.04 nmol/L) relative to the control group (182.98 ± 2.89 nmol/L). Cytochrome P450 (CYP450) activity remained similar across all groups, with the SCY group showing the lowest activity (145.74 ± 1.72 U/mL).

### 2.11. Effects on Population Dynamics of PWN

Population parameters of PWN under different treatments are summarized in Table 4. The control group exhibited the highest population counts (3751.00 ± 632.03), adult counts (1938.45 ± 403.90), and larval counts (1812.56 ± 244.24) among all group. The SCY group induced substantial population suppression, reducing the total population, adult, and larval counts by 70.83%, 70.64%, and 71.03%, respectively. The SCA group demonstrated stronger suppression, with an 84.55% reduction in adult counts. The SCF group exhibited the strongest inhibitory effect, with a total population of 143.60 ± 14.63, accounting for only 3.83% of the control, along with significantly reduced larval (59.93 ± 9.20) and adult counts (83.67 ± 10.66).

Analysis of developmental stage revealed no significant differences in adult-to-larva ratios among groups. Sex ratio analysis showed a female-to-male ratio of 3.55 ± 0.91 in the control group. All treatments significantly increased the proportion of males (SCY: 0.84 ± 0.38; SCA: 0.28 ± 0.09; SCF: 0.95 ± 0.36). Males in the SCF group exhibited the longest body length (762.07 ± 22.58 μm), followed by those in the SCA group (739.82 ± 24.36 μm), control group (709.77 ± 25.36 μm), and SCY group (677.13 ± 21.09 μm). The body length of females showed no statistical differences across treatments.

### 2.12. Differentially Expressed Genes (DEGs) Analysis of PWN

**Figure 3:**
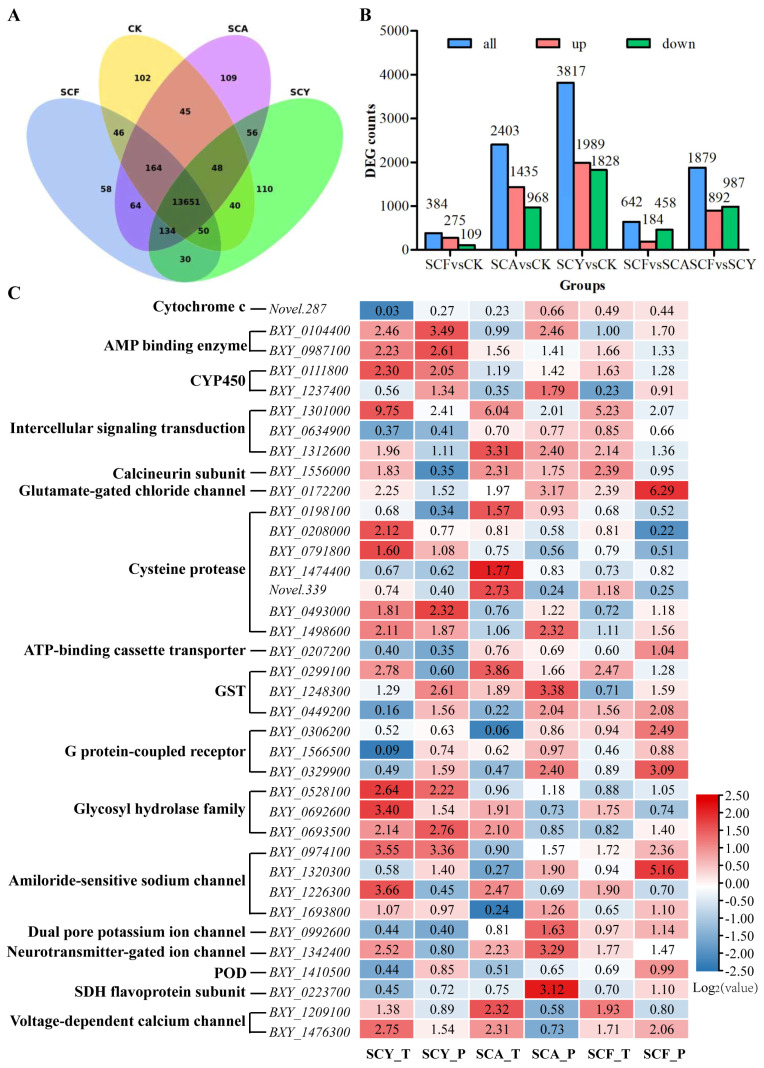
Gene expression analysis of PWN after different treatments. (**A**) Co-expression Venn diagram. (**B**) Differentially expressed gene counts. (**C**) Heatmap of transcriptomic analysis (T) vs. qRT-PCR validated genes (P) under different treatments (vs. control). SCY_T, SCA_T, and SCF_T for transcriptome data; SCY_P, SCA_P, and SCF_P for qRT-PCR data; The fold change and relative expression values are displayed within the heatmap.

High-throughput transcriptome sequencing of treated PWN samples yielded 6.93–7.60 Gb of raw bases and 6.54–7.34 Gb of high-quality clean bases (Table S1). A Venn diagram (Figure 3A) revealed 13,651 constitutively expressed genes across all treatment groups. The control, SCY, SCA, and SCF groups exhibited 102, 110, 109, and 58 unique genes, respectively. Pairwise comparisons revealed 40 (SCY vs. control), 45 (SCA vs. control), and 46 (SCF vs. control) overlapping genes.

DEGs were selected (|log_2_Fold Change (FC)| ≥ 1 and false discovery rate (FDR) ≤ 0.05), and specific expression patterns were identified across the different treatments (Figure 3B). The SCY vs. control comparison showed the most substantial transcriptomic alterations, with 3817 DEGs (1989 upregulated and 1828 downregulated). In contrast, the SCF vs. control comparison yielded only 384 DEGs (275 upregulated and 109 downregulated). Comparative analysis identified 642 DEGs between SCF and SCA, and 1879 DEGs between SCF and SCY.

Gene Ontology (GO) analysis of DEGs in PWN under different treatments is presented in Figure S1. Compared to the control, the SCF group exhibited significant alterations across 163 biological processes, 52 cellular components, and 137 molecular functions. Functional annotation revealed predominant enrichment in zinc ion binding (GO:0008270) and transcription factor activity (GO:0000988; GO:0000989; GO:0003712). Additionally, cellular compartment analysis indicated strong representation of non-membrane-bounded organelle structures (GO:0043228) and intracellular membrane- bounded organelles (GO:0043231).

In the SCA vs. control comparison, 310 biological processes, 100 cellular components, and 238 molecular functions were affected. SCA treatment upregulated genes associated with structural molecules, particularly in organelles (GO:0044446; GO:0044422; GO:0031967) and cell membranes (GO:0031090). Enhanced expression was also observed in genes related to peptide metabolism (GO:0006518) and amide biosynthesis genes (GO:0043603). Similarly, the SCY vs. control comparison identified 312 biological processes, 98 cellular components, and 248 molecular functions, with marked enrichment of genes linked to structural molecules (GO:0005198), peptide metabolism (GO:0006518), amide biosynthesis (GO:0043604), ribosomal structure (GO:0003735), and protein synthesis (GO:0065003). In the SCF vs. SCA comparison, structural molecule activity (GO:0005198) and transcription factor activity (GO:0000988; GO:0000989) were significantly enhanced, with a notable increase in acyltransferase- related genes (GO:0016407; GO:0016410; GO:0008080). In the SCF vs. SCY comparisons, the addition of abamectin upregulated genes involved in macromolecule biosynthesis (GO:0010556), oxidative stress response (GO:0006979), and membrane/organelle synthesis (GO:0043227; GO:0043231).

Kyoto Encyclopedia of Genes and Genomes (KEGG) pathway enrichment analysis of DEGs under different treatments is shown in Figure S2. Compared to the control, the SCF group displayed significant enrichment in 53 pathways, with prominent enrichment in drug metabolism-CYP450 (cel00982), longevity regulating pathway (cel04213), ATP- dependent chromatin remodeling (cel03082), and ABC transporter (cel02010).

The SCA group exhibited enrichment in 111 pathways compared to the control, including the transforming growth factor-β (TGF-β) signaling pathway (cel04350), oxidative phosphorylation (cel00190), citrate cycle (Tricarboxylic acid cycle, TCA cycle) (cel00020), and adenosine triphosphate (ATP)-dependent chromatin remodeling (cel03082). The SCY group showed enrichment in 114 pathways, with significant enrichment in TGF-β signaling pathway (cel04350), oxidative phosphorylation (cel00190), ATP-dependent chromatin remodeling (cel03082). Notably, the SCY treatment specifically activated the ErbB signaling pathway (map04012). Comparative analysis of SCF vs. SCA and SCY treatments revealed significant enrichment of SCF-responsive genes in critical pathways, including mammalian target of rapamycin (mTOR) signaling pathway (cel04150), mitogen-activated protein kinase (MAPK) signaling pathway (map04010), calcium signaling pathway (map04020), longevity regulating pathway (cel04213), Forkhead box O (FoxO) signaling pathway (cel04068), citrate cycle (cel00020), drug metabolism-other enzymes (cel00983), and ATP-dependent chromatin remodeling (cel03082). These findings indicate that the abamectin-rotenone combination demonstrates pronounced synergistic effects through the simultaneous disruption of the PWN’s material metabolism, signal transduction systems, and energy synthesis pathways.

### 2.13. Gene Expression Validation of DEGs in PWN

To investigate the potential mechanism, 37 DEGs related to drug targets, growth metabolism, and signal transduction were analyzed by quantitative real-time polymerase chain reaction (qRT-PCR) (Figure 3C and Table S2). The majority of genes showed expression trends consistent with the transcriptome analysis. For example, genes encoding glutamate-gated chloride channels (*BXY_0172200*), amiloride-sensitive sodium channel (*BXY_0974100*), CYP450 (*BXY_0111800*), intercellular signaling transduction (*BXY_1301000*, *BXY_1312600*), AMP-binding enzymes (*BXY_0104400*, *BXY_0987100*), and neurotransmitter-gated ion channel (*BXY_1342400*) were all significantly upregulated in response to SCY, SCA, and SCF treatments. In contrast, genes for Cyt C (*novel.287*), POD (*BXY_1410500*), intercellular signaling transduction (*BXY_0634900*), G protein-coupled receptors (*BXY_1566500*), and ATP-binding cassette transporter (*BXY_0207200*) were all significantly downregulated. Furthermore, SCY, SCA, and SCF treatments inhibited the expression of genes encoding cysteine proteases (*BXY_0198100*, *BXY_0208000*, *BXY_0791800*, *BXY_1474400*, *novel.339*), and glycosyl hydrolases (*BXY_0692600*), whereas the expression of genes for GST (*BXY_0299100*, *BXY_1248300*, *BXY_0449200*), and cysteine proteases (*BXY_0493000*, *BXY_1498600*) was enhanced. However, several genes exhibited expression trends by qRT-PCR that differed from those in the transcriptome analysis, including genes for SDH flavoprotein subunit (*BXY_1209100*), amiloride-sensitive sodium channel (*BXY_1320300*, *BXY_1226300*), and G protein- coupled receptor (*BXY_0329900*). Among these, the expression of *BXY_1209100* and *BXY_1226300* decreased in all SCY, SCA, and SCF treatments according to qRT-PCR, while that of the other two increased. Genes from the glycosyl hydrolase family (*BXY_0528100*, *BXY_0692600*, *BXY_0693500*), and the voltage-dependent calcium channel (*BXY_1476300*) were upregulated primarily in the SCY groups, whereas genes for SDH flavoprotein subunit (*BXY_0223700*), dual pore potassium ion channel (*BXY_0992600*), and G protein-coupled receptor (*BXY_0306200*) were downregulated. In the SCA group, the gene for calcineurin subunit (*BXY_1556000*) was significantly upregulated, while that for G protein-coupled receptor (*BXY_0306200*) was significantly downregulated. Additionally, SCF treatment significantly increased the expression of the voltage-dependent calcium channel (*BXY_1476300*).

## 3. Discussion

PWD, a devastating threat to global pine forests, urgently requires effective and sustainable control strategies [1,2,3,4]. Botanical active ingredients offer significant advantages for green pest management due to their eco-friendliness, low residue levels, and reduced risk of resistance development [21]. This study introduced a synergistic combination of the botanical compound rotenone with the chemical pesticide abamectin against the PWN. The abamectin-rotenone combination (SCF) achieved a CTC of 231.09 and an LC_50_ of 3.18 mg/L, demonstrating significantly higher efficacy than abamectin alone (LC_50_ = 8.18 mg/L) or rotenone alone (LC_50_ = 5.94 mg/L). The SCF treatment maintained egg production at 2.60 ± 0.25 eggs/female and inhibited the hatching rate to 13.09% ± 0.02% at 60 h. Head oscillation behavior was almost completely suppressed, and the total nematode population decreased to only 3.83% of that in the control group after 24 h. In related research on PWN control, a 1.8% abamectin·4.2% flupyradifurone formulation exhibited an LC_50_ of 2.4 mg/L [23]. A 2% abamectin·6% fluopyram formulation achieved an LC_50_ of 2.0208 mg/L and inhibited the hatching rate to 20.32% after a 24 h treatment at 3 mg/L [24]. A fluopyram:emamectin benzoate (3:5) mixture (LC_50_ = 3.2340 mg/L) reduced egg production to 21.00 ± 2.00 eggs/female and the hatching rate to 28.78% over 30 h [26]. A 10% fluopyram·2% abamectin formulation (LC_50_/36 h = 0.0585 mg/L) resulted in 6.80 ± 0.47 eggs/female at 0.0204 mg/L [27]. Thus, the abamectin-rotenone combination demonstrates indoor control efficacy comparable to that of these reported chemical pesticide complexes in laboratory settings, with indications of potentially longer-lasting effects based on the sustained suppression observed.

Additionally, the SCF formulation proved effective in controlling PWD symptoms when sprayed at a concentration 400-times the synergistic concentration (2.29 g/L abamectin + 0.71 g/L rotenone) on *P. massoniana* seedlings at both 7 dpi and 14 dpi. These results are consistent with a previously reported study on an abamectin-rotenone combinations targeting *Pieris rapae* on cabbage plants, which highlighted its high efficacy, rapid action, and field safety [28]. The botanical origin of rotenone could enhance its suitability for use in ecologically sensitive areas or for pine forest management, addressing environmental concerns associated with high chemical usage in conventional strategies. Significant efforts have been made to improve the performance of abamectin, particularly to address its poor water solubility and rapid photolysis. For example, new analogs of avermectin have been synthesized to achieve stronger nematicidal activity against PWN [29,30,31]. Moreover, various nanopesticide formulations have been designed to enhance the bioactivity of abamectin. For instance, abamectin@BSA nanoparticles exhibited excellent sustained-release properties, with continuous release for approximately 20 days, along with a 35.3% increase in stomach toxicity and a 19.6% increase in contact toxicity [32]. Avermectin nanoparticles composed of poly-γ-glutamic acid and chitosan achieved a mortality rate of 98.6% after 24 h at 1 ppm and were found to accumulate in the intestines and heads of nematodes [33]. The porous-structured abamectin@CuBTC had been proposed as an environmentally friendly nanopesticide that efficiently delivered abamectin to larval intestines for absorption. This formulation can be transmitted to epidemic areas and dead wood at low concentrations (10 mg/L) [17]. In a recent study, xylene was demonstrated to be an effective agent in nematode control formulations. The combination of xylene and abamectin achieved a 100% mortality rate at a significantly lower concentration of 0.5 µg/mL. Furthermore, it effectively inhibited the development of PWD in 5-year-old *P. densiflora* trees at a dosage of 0.45 mg per tree under greenhouse conditions [20]. Therefore, it is essential to improve the field applicability and stability of the abamectin-rotenone combination by integrating adjuvants or nanocarriers in future studies and practical applications.

It is known that abamectin primarily disrupts neurophysiological activity by enhancing the activity of glutamate-gated chloride channels. It promotes the release of GABA, increases chloride ion influx, hyperpolarizes the cell membrane, and inhibits action potential generation, thereby blocking nerve conduction in arthropods. This ultimately leads to muscle paralysis due to impaired contraction [34,35,36]. Rotenone, a mitochondrial respiratory inhibitor, binds to the ubiquinone-binding site of Complex I (Nicotinamide adenine dinucleotide—hydrogen (reduced) dehydrogenase, NADH dehydrogenase) in the mitochondrial electron transport chain. This binding prevents electron transfer from NADH to ubiquinone, inhibits the intracellular electron transport chain, and reduces ATP levels. The resulting insufficient energy supply impairs the function of detoxification enzyme systems. Concurrently, rotenone induces increased reactive oxygen species (ROS) production, mitochondrial Ca^2+^ overload, and opening of the mitochondrial permeability transition pore (mPTP), triggering cellular damage or apoptosis. These effects manifest as sluggish movement, paralysis, and eventual slow death [37,38,39,40]. Collectively, these actions result in decreased movement, vitality, and population size, since head oscillation, feeding, and reproductive behavior of PWN were all suppressed by either abamectin or rotenone. The synergistic combination demonstrated enhanced effects attributable to the combination of different mechanisms: inhibition of neurological activity and obstruction of energy generation. For example, the abnormal elevation in the activity of Cyt C and SDH indicates TCA cycle obstruction and ATP synthesis failure due to mitochondrial dysfunction [41,42]. Moreover, increased POD and GST activity suggested potential disruption of the oxidative stress and detoxification system by SCF [19]. Reduced ATP supply directly impaired energy requirements for egg-laying, embryonic development and other behaviors [39]. The detoxification process consumes large amounts of NADPH and GSH, leading to a diversion of energy (ATP) and reducing power resources, which may inhibit nematode growth and reproduction. Although PWN induced CYP450 genes (e.g., *BxCYP33C9*) to accelerate abamectin hydroxylation [43], rotenone-induced ATP deficiency rendered CYP450-dependent oxidation unsustainable. This disrupted drug metabolism pathways and caused toxic accumulation [38,44]. This mechanism parallels the CYP-35 plasticity observed in *C. elegans* responding to polycyclic aromatic hydrocarbons [45]. However, except for GST related genes (*BXY_0299100*, *BXY_1248300*, *BXY_0449200*), the expression trends of the other four enzymes showed significant divergence between mRNA expression levels and enzymatic activity changes. An elevated AchE activity was also recorded, which would cause acetylcholine accumulation, nicotinic receptor overstimulation, and motor nerve blockade. These results contrast with the majority of research in which AchE was normal inhibited by active compounds. For example, *Acremonium* sp. metabolites reduce AchE activity to 0.089 U/mg [46]. This suggests the SCF might indirectly induce AChE upregulation via non-cholinergic pathways, as indicated by the significantly increased expression of glutamate-gated chloride channel (*BXY_0172200*) and neurotransmitter- gated ion channel (*BXY_1342400*) genes.

Regarding DEGS, SCF specifically activated FoxO, Wnt, calcium, and MAPK signaling pathways. FoxO coordinates antioxidant defense and energy reprogramming. Its effector, Oxidative stress-responsive serine-rich protein 1 (OSER1), enhances ROS scavenging (e.g., via GST activation) to extend lifespan [47], which is consistent with the enrichment of longevity-regulating pathway. Under pesticide stress, the FoxO transcription factor DAF-16 inhibits glycolysis genes (e.g., *pdk-1*) to promote fatty acid β-oxidation (which provides ATP/NADPH for detoxification) while simultaneously activating autophagy genes (e.g., *lgg-1*) to maintain proteostasis [48,49]. The strong activation of FoxO by SCF underscores rotenone’s role in inhibiting energy synthesis. Wnt signaling establishes detoxification networks through crosstalk with FoxO. The Wnt/β-catenin cascade, BAR1–POP1 axis, directly activates transcriptional factor DAF-16 to co-induce the expression of antioxidant genes (*sod-3*, *ctl-1*) and phase II detoxification enzymes (e.g., GSTs) [50]. BAR-1 binding to the DAF-16 promoter enhances its transcriptional activity, while inhibition of glycogen synthase kinase-3 (GSK-3) amplifies antioxidant responses. Wnt signaling also strengthens defense through the cholinergic immune axis: pesticide-mediated inhibition of AChE causes acetylcholine accumulation, which activates intestinal muscarinic receptors (GAR-2/GAR-3) to induce the Wnt ligand CWN-2, thereby driving the expression of antimicrobial peptide *clec-60* [51]. The enrichment of calcium signaling pathways in the SCF groups suggests that abamectin may target calcium channels, such as ryanodine receptors (RyRs) and voltage-gated calcium channels (VGCCs). Analogous mechanisms are observed with other pesticides: diamide insecticides bind to the phosphorylation domains of RyR, causing calcium overload and muscle contraction [52], while pyrethroids target L-type VGCCs to disrupt neuronal activity, with resistance often involving altered channel kinetics [53]. This potential synergy could enhance PWN control efficacy. MAPK pathway upregulation may alleviate oxidative damage through the regulation of antioxidants [54], but its sustained activation can trigger programmed cell death [55]. Moreover, SCF treatment also induced the aberrant upregulation of the G protein-coupled receptor genes (e.g., *BXY_0306200*, *BXY_0329900*), which is predicted to interfere with chemotaxis and avoidance behaviors that are critical for host location and feeding initiation [19].

In a recent study, rotenone was found to reduce the lifespan and muscle activity of wild-type *Caenorhabditis elegans* nematodes. It increased ROS production, decreased mitochondrial membrane potential, and disrupted mitochondrial ultrastructure. However, CGP37157, a mitochondrial Na^+^/Ca^2+^ exchanger inhibitor, partially or completely reversed most of these alterations [56]. Our study revealed DEG enrichment in functions such as metal ion binding, and gene expression analysis confirmed the significant upregulation of genes associated with glutamate-gated chloride channels (*BXY_0172200)*, voltage-dependent calcium channels (*BXY_1476300*), amiloride-sensitive sodium channels (*BXY_0974100*, *BXY_1320300*), intercellular signaling transduction (*BXY_1301000*, *BXY_1312600*), and neurotransmitter-gated ion channels (*BXY_1342400*). Thus, we propose a hypothesis that rotenone induces Na^+^/Ca^2+^ imbalance, thereby enhancing the Cl^−^ influx triggered by abamectin and resulting in an “Na^+^/Ca^2+^/Cl^−^ ionic storm.” This storm may lead to oxidative stress, osmotic disruption, and impaired energy metabolism, ultimately causing cell death. From another perspective, as mentioned above, abamectin increases chloride ion influx, while rotenone causes mitochondrial Ca^2+^ overload. Moreover, rotenone also activates voltage-dependent calcium channels, causing a surge in cytosolic Ca^2+^ influx. The inhibition of ATP synthesis can further impair Na^+^/K^+^-ATPase function, resulting in Na^+^ accumulation and K^+^ efflux. This reversal of the Na^+^/Ca^2+^ exchanger operation promotes additional Ca^2+^ influx and calcium overload, ultimately inducing membrane depolarization [37,38,39,40]. Consequently, these two compounds may establish a synergistic cascade mechanism: “Na^+^ influx → aggravated Ca^2+^ overload → activation of Cl^−^ channels → Cl^−^ efflux → accelerated Na^+^ influx.” This cascade disrupts intracellular ion homeostasis, exacerbates oxidative stress, compromises plasma membrane integrity, and potentially leads to the death of PWN cells. However, several key questions warrant further investigation: Do the roles of Cl^−^, Na^+^, and Ca^2+^ exhibit temporal changes and interactions? Is there a potential division of labor where Cl^−^ initiates the process, Ca^2+^ acts as the core driver, and Na^+^ serves to amplify the effect? What roles do core cellular organelles (e.g., mitochondria) and intracellular calcium stores play? Are oxidative stress and energy deficiency the cause or the consequence of the ion imbalance? Is the cell death induced by the combined treatment primarily apoptosis or necrosis? Does a competitive relationship exist, where low concentrations favor apoptosis and high concentrations lead to necrosis? Addressing these questions would deepen the understanding of the mechanisms of the rotenone-abamectin combination and promote the development of eco-friendly preventive agents for PWN.

In summary, this study identified a synergistic combination of abamectin and rotenone with potent nematicidal activity against PWN. The co-formulation exerts its synergistic lethality through multi-target mechanisms, including enhanced drug retention, inhibition of key physiological behaviors (e.g., reproduction and feeding), disruption of energy metabolism, amplification of oxidative stress signals, and interference with detoxification enzyme systems. These findings provide a robust foundation for developing novel chemical control strategies and offer a green, efficient solution for the management of PWD.

## 4. Materials and Methods

### 4.1. Nematode Source and Cultivation

The PWN strain (SGSX) was originally isolated from Shixing, Shaoguan, China, in 2019. The strain is preserved at Guangdong Provincial Key Laboratory of Silviculture, Protection and Utilization, Guangdong Academy of Forestry, Guangzhou, China. Nematodes were maintained on potato dextrose agar (PDA) plates inoculated with *Pestalotiopsis* spp. and cultured at 25 °C. The PDA medium was prepared using potato dextrose powder (Guangdong Huankai Biotechnology, Zhaoqing, China) and agar powder (Beijing Dingguo Changsheng Biotechnology, Beijing, China).

### 4.2. Indoor Toxicity Testing

The culture plates were washed with sterile water after 3–4 days of PWN feeding, and the nematode suspension was adjusted to a density of approximately 100 nematodes/100 μL. Stock solutions (10 mg/mL) of each compound (Detailed information is provided in Table S3) were prepared in appropriate solvents and serially diluted to concentrations of 1000, 500, 200, 100, and 50 mg/L. Subsequently, 1 μL of each test reagent dilution was mixed with 99 μL of the nematode suspension in 48-well plates (NEST Biotechnology, Wuxi, China), resulting in final test concentrations of 10, 5, 2, 1, and 0.5 mg/L. The corresponding solvent was used as the control. The solvent of curcumin was acetone and for ethyl allicin it was sterile water, while DMSO was the solvent for the other compounds. Each treatment had three technical replicates, with at least two biological repeats. Nematode mortality was assessed after 24 h of exposure. The corrected mortality rate, toxicity regression equations, LC_50_, correlation coefficient (R), and 95% confidence intervals were calculated according to the method described by Zhang et al. [57]. The formulas are as follows:

Mortality (%) = (Number of dead nematodes/Total number of nematodes) × 100%

Corrected mortality (%) = [(Mortality of treated group − Mortality of control group)/(1 − Mortality of control group)] × 100%

### 4.3. Interaction Assay

Abamectin, emamectin benzoate, and fluopyram were tested in combination with rotenone at ratios partitioned from their individual LC_50_ values. Each treatment had three technical replicates with two biological trials. The formulas are as follows:

Expected mortality (%) = (Mortality contribution of component A × Proportion of A) + (Mortality contribution of component B × Proportion of B)

Toxicity ratio = Actual mortality/Expected mortality

### 4.4. CTC Method

The top three mixing ratios of synergistic combinations of abamectin-rotenone and emamectin benzoate-rotenone were selected for CTC analysis [58], respectively. The formulas are as follows:

Toxicity index (TI) = (LC_50_ of standard component/LC_50_ of tested component) × 100

Actual toxicity index (ATI) = (LC_50_ of standard component/LC_50_ of mixture) × 100

Theoretical toxicity index (TTI) = (TI of A × Proportion of A) + (TI of B × Proportion of B)

CTC = (ATI/TTI) × 100

### 4.5. Pot Experiment

The most effective synergistic combination of abamectin-rotenone was selected for further experiments with four treatments, including abamectin (5.73 mg/L, SCA), rotenone (1.78 mg/L, SCY), the synergistic combination (abamectin 5.73 mg/L + rotenone 1.78 mg/L, SCF) and a solvent control (DMSO, ≤1%, CK).

A 1 mL PWN suspension (about 1000 nematodes) was inoculated into a stem incision made at 20 cm above the base of each three-year-old *P. massoniana* seedling, and a moist cotton ball was sealed with cling film to maintain humidity. The 50-, 100-, 200-, and 400-times concentrations of reagent solutions (control, SCA, SCY, SCF) were prepared using 0.10% Toucui penetrating agent (Shenzhen Noposion Crop Science, Shenzhen, China). The corresponding final concentrations of abamectin and rotenone in the spray solutions were 286.50 & 89.00, 573.00 & 178.00, 1146.00 & 356.00, and 2292.00 & 712.00 mg/L, respectively. Treatments were applied via foliar spray at 7 and 14 days post-inoculation. Disease progression was monitored weekly for 60 days.

### 4.6. Morphological Observation

Nematode suspensions were treated with the control, SCA, SCY, and SCF solutions for 48 h in the dark. A 50 μL nematode suspension of each treatment was transferred onto a glass slide, and morphological observation was conducted using a fluorescence inverted microscope (Vert.A1, ZEISS, Oberkochen, Germany). There were five technical replicates of each treatment and at least two biological replicates.

### 4.7. Feeding Assay

A nematode suspension (approximately 3000 nematodes) was treated with the control, SCA, SCY, and SCF solution for 24 h. The nematodes were then centrifuged at 2000 rpm for 5 min, washed three times with sterile water, and transferred onto PDA plates fully-covered with *Pestalotiopsis* spp. The plates were incubated at 25 °C in darkness and fungal consumption was recorded every 3 days. Each treatment included five replicates, with at least two biological repeats.

### 4.8. Head Thrashes Assay

Nematode suspensions (approximately 100 nematodes) were treated with the control, SCA, SCY, and SCF solutions. Ten nematodes were randomly selected at 12, 24, 36, 48, and 60 h post-treatment and recorded the number of head thrashes per minute under a microscope. Each treatment included five replicates, with at least two biological repeats. The thrashing inhibition rate was calculated as follows:

Thrashing inhibition rate (%) = [(Control thrash count − Treatment thrash count)/Control thrash count] × 100.

### 4.9. Fecundity Detection

Ten female and ten male adults were transferred into a 48-well plates and treated with the control, SCA, SCY, and SCF solutions in the dark. The number of eggs laid was recorded at 12, 24, 36, 48, and 60 h. Each treatment included three replicates, with at least two biological repeats. The calculation formula is as follows:

Average egg production per female = Total number of eggs/10.

### 4.10. Egg Hatching Assay

A 1 mL suspension of mixed-stage nematodes (approximately 1000 nematodes) was transferred into a 48-well plate and incubated at 25 °C in darkness for 12 h. The eggs adhering to the bottom of the wells were then washed 3 times with sterile water. After treatment with the control, SCA, SCY, and SCF solutions for 12, 24, 36, 48, and 60 h, the hatching rate was determined. Each treatment included three replicates, with at least two biological repeats. The calculation formula is as follows:

Egg hatching rate (%) = [Number of larvae/(Number of eggs + Number of larvae)] × 100%.

### 4.11. Enzyme Activity Assay

Approximately 10,000 nematodes were treated with the control, SCA, SCY, and SCF solutions for 24 h. The nematodes were collected by centrifugation, washed three times, and homogenized on ice using a sample grinder (JXFSIPRP-CL, Shanghai Jingxin Industry, Shanghai, China) at 60 Hz for 40 s. The homogenate was centrifuged at 8000 rpm and 4 °C for 10 min, and the supernatant was collected for enzyme activity assays according to the instructions for the commercial kits of POD, SDH, AchE, GST, CYP450, and Cyt C (Shanghai Enzyme-linked Biotech, Shanghai, China). Each treatment included three replicates, with at least two biological repeats.

### 4.12. Population Dynamics Detection

After treating with the control, SCA, SCY, and SCF solutions for 24 h, the nematodes suspensions were serially diluted to determine the total number of nematodes, adults, larvae, females, and males using an optical microscope. Ten randomly selected female and male adults were heat-killed to measure their body lengths under an inverted microscope. Each treatment included five replicates, with at least two biological repeats.

### 4.13. Transcriptomic Analysis

Approximately 20,000 mixed-stage nematodes were treated with the control, SCA, SCY, SCF solutions for 24 h. The nematodes were then collected at 5000 rpm for 5 min and immediately flash-frozen in liquid nitrogen. All subsequent transcriptomic sequencing and preliminary analyses were performed by Novogene (Beijing, China).

Briefly, total RNA integrity and quantity were assessed using an Agilent 2100 bioanalyzer (Agilent Technologies, Santa Clara, CA, USA). Strand-specific RNA-seq libraries were constructed and sequenced on an Illumina NovaSeq 6000 platform (Illumina, San Diego, CA, USA) to generate 150-bp paired-end reads. Raw sequencing reads were processed with fastp (v0.23.2) to remove adapter-contaminated reads, poly-N sequences, and low-quality reads (Q20 < 90%). The clean reads were aligned to the reference genome of *Caenorhabditis elegans* using HISAT2 (v2.0.5) with default parameters. Gene expression levels were quantified using featureCounts (v1.5.0-p3). Read counts for each gene were normalized to FPKM (Fragments Per Kilobase of transcript per Million mapped reads) to account for gene length and sequencing depth differences. Biological replicate datasets were analyzed via DESeq2 using negative binomial models, applying dispersion shrinkage and Benjamini–Hochberg FDR correction (Padj ≤ 0.05). The edgeR performed precision-weighted analysis with TMM normalization and empirical Bayes dispersion estimation, selecting DEGs at |log_2_FC| ≥ 1 and FDR ≤ 0.05. GO enrichment analysis and functional annotation were conducted using clusterProfiler (v3.18.1) with gene length bias correction. Terms with Padj < 0.05 were considered significantly enriched. KEGG pathway enrichment analysis was performed to identify metabolic and signaling pathways associated with DEGs using clusterProfiler (v3.18.1).

### 4.14. qRT-PCR Validation of DEGs

After treatment with the control, SCA, SCY, and SCF solutions for 24 h, nematodes were collected. Total RNA was isolated using Trizol reagent (Takara Bio, Beijing, China) following the standard procedure. RNA concentration and purity were measured using a full-wavelength spectrophotometer (OSE-260-06, Tiangen Biotech, Beijing, China). Then cDNA was synthesized following the manufacturer’s protocol of PrimeScript™ II 1st strand cDNA synthesis kit (Takara Bio, Beijing, China). Primers were designed using Primer Premier 5.0 (Premier Biosoft International, San Francisco, CA, USA) and synthesized by Qingke Biotech (Guangzhou, China). Detailed primer sequences are listed in Table S4. A 10 μL reaction mixture, containing 5 μL of 2× TB Green Premix Ex Taq II Fast qPCR Mix (Takara Bio, Beijing, China), 0.5 μL of each forward and reverse primer, 0.2 μL of cDNA template and 3.8 μL of RNase-free water, was carried out following the thermal cycling conditions: 95 °C for 3 min; 39 cycles of 95 °C for 10 s, annealing temperature (Tm) for 10 s, 72 °C for 10 s; followed by a melt curve analysis. The relative expression levels of target genes were normalized to the reference gene *BX-Actin* using the 2^−*ΔΔ*Ct^ method. Each experiment included three technical replicates and three biological replicates. A heatmap of gene expression was generated by TBtools-II (v2.333) [59] with log2-transformed and row-scaled normalized expression values.

### 4.15. Statistical Analysis

Data were organized using WPS Office 2023 (Kingsoft Office, Beijing, China). LC_50_ values and their 95% confidence intervals were determined by probit regression analysis. Statistical significance testing was performed using one-way ANOVA followed by Duncan’s test in SPSS Statistics 26 (IBM, Armonk, NY, USA). Data visualization and graph generation were performed with GraphPad Prism 8.0.2 (GraphPad Software, San Diego, CA, USA).

## Figures and Tables

**Table 1:** Nematicidal activity of chemical and biogenic active ingredients against PWN.

Ingredients	Toxicity Regression Equation	R^2^	LC_50_ (mg/L)	95% Confidence Interval
Fluopyram	y = 2.4312 x + 4.2498	0.9948	2.25	1.86–2.71
Emamectin benzoate	y = 1.8536 x + 3.8069	0.9983	5.98	4.38–8.16
Abamectin	y = 2.1610 x + 3.3494	0.9909	8.18	6.05–11.06
Fluensulfone	y = 5.0938 x − 9.6708	0.9695	817.97	726.09–921.49
Fosthiazate	y = 1.6539 x − 0.5744	0.9945	2137.03	528.40–8642.82
Thiamethoxam	y = 0.8737 x + 1.8904	0.9775	5173.82	181.19–∞
Chlorantraniliprole			>40.00	
Tebufenozide			>10.00	
Rotenone	y = 1.3234 x + 3.7864	0.9340	5.94	4.52–7.81
Harmine	y = 1.1354 x + 2.8432	0.9992	68.07	49.42–93.75
Curcumin	y = 1.5365 x + 2.1590	0.9812	80.05	20.12–318.43
Spermidine	y = 1.2283 x + 2.4979	0.9965	87.82	54.95–140.35
Ethyl allicin	y = 3.3957 x − 1.5983	0.9827	91.08	83.34–99.53
Camptothecin	y = 0.7343 x + 3.2589	0.9762	104.15	5.08–2135.70
Osthole	y = 1.8298 x + 0.8462	0.9807	164.98	116.09–234.46
Chitosan	y = 1.8199 x + 0.4786	0.9957	331.93	192.91–571.12
Norcantharidin	y = 1.5716 x + 0.3204	0.9043	767.26	190.53–3089.72
2-Oxobutyric acid	y = 1.6370 x − 0.0597	0.9841	1028.85	678.53–1560.04
Azadirachtin	y = 4.5132 x − 9.3308	0.9520	1639.23	1479.26–1816.51
Matrine	y = 2.3353 x − 2.5480	0.9175	1718.56	740.16–3990.25

Note: For chlorantraniliprole and tebufenozide, precipitation occurred at high concentrations and their LC_50_ values are expressed as greater than the maximum observed concentration. The solvent of curcumin was acetone and for ethyl allicin it was sterile water, while dimethyl sulfoxide was the solvent for the other compounds.

**Table 2:** Nematicidal activity of compound combinations with rotenone against PWN.

Group	1	2	3	4	5	6	7	8	9	10	11
Formulation Ratio	10:0	9:1	8:2	7:3	6:4	5:5	4:6	3:7	2:8	1:9	0:10
Emamectin benzoate (mg/L)	5.98	5.38	4.78	4.19	3.59	2.99	2.39	1.79	1.20	0.60	0.00
Rotenone (mg/L)	0.00	0.59	1.19	1.78	2.38	2.97	3.56	4.16	4.75	5.35	5.94
Actual mortality (%)	44.99	46.21	62.42	72.46	68.42	67.04	58.25	63.19	63.75	73.67	53.58
Expected mortality (%)	44.99	45.85	46.71	47.57	48.43	49.29	50.14	51.00	51.86	52.72	53.58
Toxic ratio	1.00	1.01	1.34	1.52	1.41	1.36	1.16	1.24	1.23	1.40	1.00
Abamectin (mg/L)	8.18	7.36	6.54	5.73	4.91	4.09	3.27	2.45	1.64	0.82	0.00
Rotenone (mg/L)	0.00	0.59	1.19	1.78	2.38	2.97	3.56	4.16	4.75	5.35	5.94
Actual mortality (%)	44.68	52.45	71.28	79.17	80.08	78.49	79.53	74.97	70.72	71.39	52.26
Expected mortality (%)	44.68	45.44	46.20	46.95	47.71	48.47	49.23	49.99	50.74	51.50	52.26
Toxic ratio	1.00	1.15	1.54	1.69	1.68	1.62	1.62	1.50	1.39	1.39	1.00
Fluopyram (mg/L)	2.25	2.03	1.80	1.58	1.35	1.13	0.90	0.68	0.45	0.23	0.00
Rotenone (mg/L)	0.00	0.59	1.19	1.78	2.38	2.97	3.56	4.16	4.75	5.35	5.94
Actual mortality (%)	47.85	46.52	48.71	52.34	53.71	56.77	46.98	52.55	41.85	49.88	53.81
Expected mortality (%)	47.85	48.45	49.04	49.64	50.23	50.83	51.43	52.02	52.62	53.21	53.81
Toxic ratio	1.00	0.96	0.99	1.05	1.07	1.12	0.91	1.01	0.80	0.94	1.00

Note: Synergism: Toxicity ratio > 1.25; Antagonism: Toxicity ratio < 0.75; Additivity: 0.75 < toxicity ratio < 1.25.

**Table 3:** CTC methods analysis of compound combination against PWN.

Compounds	Ratio	Toxicity Regression Equation	R^2^	LC_50_ (mg/L)	95% Confidence Interval	CTC
Emamectin benzoate: Rotenone	7:3	y = 2.3188 x + 3.6031	0.9975	4.00	3.35–4.79	149.20
	6:4	y = 2.1317 x + 3.6905	0.9935	4.11	3.28–5.16	145.11
	5:5	y = 2.6841 x + 3.2937	0.9571	4.32	3.64–5.14	137.96
Abamectin: Rotenone	7:3	y = 2.1446 x + 3.9214	0.9110	3.18	2.55–3.98	231.09
	6:4	y = 2.0585 x + 3.8533	0.9827	3.61	2.89–4.50	196.89
	5:5	y = 1.2979 x + 4.2530	0.9684	3.76	2.66–5.33	183.04

Note: Synergism: CTC > 120; Antagonism: CTC < 80; Additivity: 80 ≤ CTC ≤ 120.

**Table 4:** Effect of different treatments on the population dynamics of PWN.

Population Index	Control	SCY	SCA	SCF
Populations	3751.00 ± 632.03 a	1094.13 ± 126.38 b	579.34 ± 82.81 b	143.60 ±14.63 b
Adults	1938.45 ± 403.90 a	569.07 ± 64.17 b	238.34 ± 35.74 b	83.67 ± 10.66 b
Larvae	1812.56 ± 244.24 a	525.07 ± 101.34 b	341.00 ± 48.32 b	59.93 ± 9.20 b
Adults: Larvae	1.61 ± 0.44 a	0.70 ± 0.04 a	1.26 ± 0.30 a	1.04 ± 0.09 a
Female	1443.45 ± 321.31 a	231.73 ± 78.08 b	47.67 ± 8.83 b	36.53 ± 8.88 b
Male	495.00 ± 116.32 a	337.33 ± 45.68 ab	190.67 ± 34.53 ab	47.13 ± 9.33 b
Female: male	3.55 ± 0.91 a	0.84 ± 0.38 b	0.28 ± 0.09 b	0.95 ± 0.36 b
Male body length/um	709.77 ± 25.36 ab	677.13 ± 21.09 b	739.82 ± 24.36 ab	762.07 ± 22.58 a
Female body length/um	841.83 ± 34.22 a	822.49 ± 21.45 a	825.40 ± 19.75 a	888.20 ± 15.06 a

Note: Different lowercase letters indicate significant differences between groups (*p* < 0.05).

## Data Availability

The original contributions presented in this study are included in the article/Supplementary material. Further inquiries can be directed to the corresponding authors.

## Supplementary Materials

The following supporting information can be downloaded at: https://www.mdpi.com/article/10.3390/ijms26189133/s1.
