# Supplementary material for: Synergistic Effects of Rotenone and Abamectin on Physiological Suppression, Population Inhibition, and Ion Disruption of Bursaphelenchus xylophilus
Source: Int J Mol Sci. 2025 Sep 18;26(18):9133. doi: 10.3390/ijms26189133 (PMC12470252; doi:10.3390/ijms26189133)
Supplement: Supplementary file 1 [file ijms-26-09133-s001.zip › Supplemental Material.pdf]

Supplemental Material

Table S1 Statistics of sequencing samples of transcriptome analysis.

Sample	Library	Raw_reads	Raw bases	Clean reads	Clean bases	Error rate	Q20	Q30	GC/%
CK1	FRAS240214568-1r	48836546	7.33G	47525716	7.13G	0.01	99.11	97.24	51.02
CK2	FRAS240214569-1r	47179862	7.08G	45058102	6.76G	0.01	99.17	97.40	51.52
CK3	FRAS240215323-1r	46188548	6.93G	44814206	6.72G	0.01	99.23	97.54	50.94
SCY1	FRAS240214567-1r	46390314	6.96G	44707848	6.71G	0.01	99.09	97.18	51.76
SCY2	FRAS240215322-1r	48379500	7.26G	46101624	6.92G	0.01	99.22	97.53	52.27
SCY3	FRAS240215324-1r	49364970	7.40G	46203350	6.93G	0.01	99.26	97.67	52.44
SCA1	FRAS240214564-1r	50426476	7.56G	47394362	7.11G	0.01	99.21	97.45	52.55
SCA2	FRAS240214565-1r	48447508	7.27G	44901054	6.74G	0.01	99.18	97.44	51.77
SCA3	FRAS240214566-1r	50680654	7.60G	48792892	7.32G	0.01	99.03	97.06	51.83
SCF1	FRAS240214562-1r	49018790	7.35G	46768508	7.02G	0.01	99.12	97.32	51.69
SCF2	FRAS240214563-1r	50307266	7.55G	48910660	7.34G	0.01	99.02	97.06	50.96
SCF3	FRAS240215321-1r	46269128	6.94G	43575538	6.54G	0.01	99.30	97.76	52.12

Table S2 Average fold change of related DEGs in PWN (vs control).

Gene	Fold change/CK		Fold change/SCY		Fold change/SCA		Fold change/SCF	
	Transcriptome	RT-qPCR	Transcriptome	RT-qPCR	Transcriptome	RT-qPCR	Transcriptome	RT-qPCR
Novel.287	1.0000	1.0062	0.0274	0.2655	0.2312	0.6588	0.4950	0.4367
BXY_0104400	1.0000	1.0313	2.4624	3.4938	0.9874	2.4557	1.0049	1.7046
BXY_0987100	1.0000	1.0313	2.2258	2.6121	1.5555	1.4146	1.6603	1.3253
BXY_0111800	1.0000	1.0000	2.2951	2.0514	1.1909	1.4161	1.6259	1.2798
BXY_1237400	1.0000	1.0485	0.5568	1.3418	0.3479	1.7872	0.2325	0.9062
BXY_1301000	1.0000	1.0243	9.7473	2.4075	6.0406	2.0142	5.2324	2.0700
BXY_0634900	1.0000	1.0050	0.3741	0.4145	0.6980	0.7680	0.8549	0.6576
BXY_1312600	1.0000	1.0135	1.9601	1.1121	3.3143	2.3991	2.1400	1.3563
BXY_1556000	1.0000	1.0971	1.8337	0.3549	2.3088	1.7524	2.3946	0.9480
BXY_0172200	1.0000	1.1813	2.2528	1.5162	1.9725	3.1713	2.3946	6.2901
BXY_0198100	1.0000	1.0113	0.6751	0.3427	1.5707	0.9334	0.6751	0.5202
BXY_0208000	1.0000	1.0220	2.1206	0.7709	0.8125	0.5814	0.8090	0.2223
BXY_0791800	1.0000	1.0257	1.6018	1.0833	0.7494	0.5626	0.7910	0.5103
BXY_1474400	1.0000	1.0597	0.6660	0.6234	1.7748	0.8342	0.7348	0.8214
Novel.339	1.0000	1.0006	0.7409	0.3989	2.7305	0.2359	1.1831	0.2457
BXY_0493000	1.0000	1.0096	1.8129	2.3205	0.7620	1.2171	0.7174	1.1764
BXY_1498600	1.0000	1.0329	2.1098	1.8676	1.0646	2.3185	1.1118	1.5622
BXY_0207200	1.0000	1.0101	0.4007	0.3461	0.7616	0.6868	0.6016	1.0406
BXY_0299100	1.0000	1.0145	2.7755	0.6011	3.8599	1.6556	2.4666	1.2774
BXY_1248300	1.0000	1.0019	1.2887	2.6055	1.8900	3.3807	0.7123	1.5896
BXY_0449200	1.0000	1.1204	0.1567	1.5607	0.2248	2.0409	1.5581	2.0807
BXY_0306200	1.0000	1.0216	0.5153	0.6269	0.0581	0.8637	0.9424	2.4900
BXY_1566500	1.0000	1.0206	0.0928	0.7420	0.6211	0.9693	0.4644	0.8840
BXY_0329900	1.0000	1.0003	0.4895	1.5945	0.4713	2.3982	0.8882	3.0897
BXY_0528100	1.0000	1.0214	2.6442	2.2234	0.9564	1.1814	0.8755	1.0527
BXY_0692600	1.0000	1.0327	3.3997	1.5392	1.9083	0.7325	1.7470	0.7399
BXY_0693500	1.0000	1.0436	2.1376	2.7639	2.0951	0.8458	0.8246	1.4037
BXY_0974100	1.0000	1.0001	3.5462	3.3631	0.9012	1.5681	1.7212	2.3596
BXY_1320300	1.0000	1.0106	0.5815	1.4017	0.2736	1.8962	0.9400	5.1585
BXY_1226300	1.0000	1.0022	3.6553	0.4511	2.4657	0.6906	1.8962	0.7013
BXY_1693800	1.0000	1.0345	1.0719	0.9710	0.2399	1.2587	0.6455	1.1031
BXY_0992600	1.0000	1.0000	0.4414	0.4028	0.8067	1.6305	0.9721	1.1395
BXY_1342400	1.0000	0.8993	2.5231	0.7988	2.2306	3.2870	1.7725	1.4705
BXY_1410500	1.0000	1.0001	0.4357	0.8497	0.5107	0.6479	0.6940	0.9948
BXY_0223700	1.0000	1.0281	0.4489	0.7157	0.7484	3.1239	0.6963	1.0997
BXY_1209100	1.0000	1.0006	1.3806	0.8877	2.3230	0.5765	1.9281	0.7954
BXY_1476300	1.0000	1.0038	2.7476	1.5381	2.3119	0.7333	1.7114	2.0646

Table S3 Detailed information of tested compounds.

Substances	CAS	Purity	State	Item code	Manufacturer
Emamectin benzoate	155569-91-8	≥ 95.00%	Solid	BD45977-25 g	Bide Pharmatech, Shanghai, China
Abamectin	71751-41-2	≥ 97.00%	Solid	SH095-25 g	Shanghai Yuanye Bio-Technology, Shanghai, China
Fluopyram	658066-35-4	≥ 98.00%	Solid	F304303-1 g	Aladdin®, Shanghai, China
Fosthiazate	98886-44-3	≥ 98.10%	Solid	71701-10 mg	TMRM Quality Inspection, Changzhou, China
Fluensulfone	318290-98-1	≥ 99.50%	Solid	722023-10 mg	TMRM Quality Inspection, Changzhou, China
Thiamethoxam	153719-23-4	≥ 97.00%	Solid	B-CC828-5g	TMRM Quality Inspection, Changzhou, China
Chlorantraniliprole	500008-45-7	≥ 95.00%	Solid	BD305835-5g	Bide Pharmatech, Shanghai, China
Tebufenozide	112410-23-8	≥ 98.00%	Solid	S31619-100g	Shanghai Yuanye Bio-Technology, Shanghai, China
Azadirachtin	11141-15-6	≈ 40.63%	Solid	-	Chengdu greengold High-Tech, Chengdu, China
Matrine	519-02-8	≥ 98.00%	Solid	M813524-25 g	Aladdin®, Shanghai, China
Camptothecin	7689/3/4	≥ 98.00%	Solid	BD38860-10 g	Bide Pharmatech, Shanghai, China
Harmine	442-51-3	≥ 98.00%	Solid	BD14622-5 g	Bide Pharmatech, Shanghai, China
Curcumin	458-37-7	≥ 98.00%	Solid	BD9137-25 g	Bide Pharmatech, Shanghai, China
Ethyl allicin	539-86-6	≥ 97.53%	Liquid	BD12996-25 g	Bide Pharmatech, Shanghai, China
Norcantharidin	29745-04-08	≥ 98.00%	Solid	D806433-25 g	Aladdin®, Shanghai, China
Oxobutyric acid	600-18-0	≥ 99.65%	Solid	BD76180-5 g	Bide Pharmatech, Shanghai, China
Chitosan	148411-57-8	≥ 98.00%	Solid	C2849-25 g	TCI (Shanghai) Development, Shanghai, China
Spermidine	124-70-9	≥ 98.00%	Solid	BD151794-5 g	Bide Pharmatech, Shanghai, China
Rotenone	83-79-4	≥ 99.00%	Solid	BD150594-5 g	Bide Pharmatech, Shanghai, China
Osthole	484-12-8	≥ 98.00%	Solid	BD7596-5 g	Bide Pharmatech, Shanghai, China

Table S4 Primer sequences for qRT-PCR.

No.	Gene name	Primers	Primer sequence (5 'to 3')	Functional annotation
1	Novel.287	Novel.287F	CTTTTAGATGCTTGTTTTGTTG	Cytochrome c
		Novel.287R	TGTGATAGCTCCTGCCAAT	
2	BXY_0104400	BXY_0104400F	GCAGTATGTTGGGTGGTG	AMP binding enzyme
		BXY_0104400R	TCCGTCCTTCGTTTGATA	
3	BXY_0987100	BXY_0987100F	ATCTAATCAAGGTGAATGGAA	AMP binding enzyme
		BXY_0987100R	GACCGAATTTAACACGACA	
4	BXY_0111800	BXY_0111800F	ACAACGACGGTGATTTATG	Cytochrome P450
		BXY_0111800R	ATGTTCCTTGATTCCCTTT	
5	BXY_1237400	BXY_1237400F	TTTGAAAGCCTTGACGAA	Cytochrome P450
		BXY_1237400R	TGTAAACTCCCTTGAAGAATG	
6	BXY_1301000	BXY_1301000F	ACAGGAAGCCAGCCGAATC	Intercellular signaling transduction
		BXY_1301000R	GCCAGCACCGAACCAAAC	
7	BXY_0634900	BXY_0634900F	CTGAGCAAAGAGCAGAAAA	Intercellular signaling transduction
		BXY_0634900R	GATCATCCTCCAGGTGTATC	
8	BXY_1312600	BXY_1312600F	AATCCCTCCATCACCCAC	Intercellular signaling transduction
		BXY_1312600R	CCTTGGACGACCAGTTTT	
9	BXY_1556000	BXY_1556000F	AGTTTTGTCGCATTTCCG	Calcineurin subunit
		BXY_1556000R	GCTCAATGTCCGTCTTCTC	
10	BXY_0172200	BXY_0172200F	TGTGGATCGGTGTTTGTAT	Glutamate-gated chloride channel
		BXY_0172200R	CGACGGGTTAGCATAGTT	
11	BXY_0198100	BXY_0198100F	CGCTCCAAGCATTCTACA	Cysteine protease
		BXY_0198100R	ATCTCCAAGGCAATCTCG	
12	BXY_0208000	BXY_0208000F	GTTCATCGTCAAGTTCGG	Cysteine protease
		BXY_0208000R	GTCAAAGTCTGGGGTATTCT	
13	BXY_0791800	BXY_0791800F	CCGACGGGAAGCAACATT	Cysteine protease
		BXY_0791800R	CCTGCGGGATACCACCAA	
14	BXY_1474400	BXY_1474400F	TCATCATCCGTTCCAAAA	Cysteine protease
		BXY_1474400R	GCTCACTCAAATCGACCA	
15	Novel.339	Novel.339F	TACAAAAGTGGCATTCTCAA	Cysteine protease
		Novel.339R	GAAATATCCCTGTTCTCCC	
16	BXY_0493000	BXY_0493000F	TTTATTTATCGGCTACGGT	Cysteine protease
		BXY_0493000R	AATCCCAGACTTGCTTTG	
17	BXY_1498600	BXY_1498600F	AGTACGACGAGGACAACG	Cysteine protease
		BXY_1498600R	TTCCACTCCATACCCAAC	
18	BXY_0207200	BXY_0207200F	TTCTTTGGGACTTGGCATC	ATP-binding cassette transporter
		BXY_0207200R	TACGGCATACGGAGGAGAT	
19	BXY_0299100	BXY_0299100F	CCACCTACGAGTTGCTGT	Glutathione S-transferase
		BXY_0299100R	TGAGGAGGACTGGGATTT	
20	BXY_1248300	BXY_1248300F	AAGGCACTTCAGCCACAT	Glutathione S-transferase
		BXY_1248300R	CAGTGCGTCAACCTTTCG	
21	BXY_0449200	BXY_0449200F	TCAATCCATCTGGGAACTT	Glutathione S-transferase
		BXY_0449200R	CAATAACCCTTACCATCATACA	
22	BXY_0306200	BXY_0306200F	AGGCGAGAATGAGGAAGT	G protein-coupled receptor
		BXY_0306200R	GAGGGTTGAACAGCGATA	
23	BXY_1566500	BXY_1566500F	CTTTGTGGCTCTAATCGG	G protein-coupled receptor
		BXY_1566500R	TTAATGAAACGGTGGTCC	
24	BXY_0329900	BXY_0329900F	AGTAACTTTTCCCATTTCGT	G protein-coupled receptor
		BXY_0329900R	GCATTTGAGGTGCTGATT	
25	BXY_0528100	BXY_0528100F	CTGGTCTCCGCAGTGAGT	Glycosyl hydrolase family
		BXY_0528100R	GCATCCGTCCTTGTTCTC	
26	BXY_0692600	BXY_0692600F	GATGGTGCTGTGCTTGCT	Glycosyl hydrolase family
		BXY_0692600R	GTCCTTCGGTGCGTTGTA	
27	BXY_0693500	BXY_0693500F	CTGTTTGTGGTGGCTTTG	Glycosyl hydrolase family
		BXY_0693500R	CGTCCTGGATTGGGTTAT	
28	BXY_0974100	BXY_0974100F	TTTTGGACAATCATCACGC	Amiloride-sensitive sodium channel

		BXY_0974100R	GCAGTCCGCATACGAGTC	
29	BXY_1320300	BXY_1320300F	TGGAAGCCTCATTCTACG	Amiloride-sensitive sodium channel
		BXY_1320300R	TTGTTGGGTCTTGATTGTC	
30	BXY_1226300	BXY_1226300F	TGGTTCCTCGTCCTTCTC	Amiloride-sensitive sodium channel
		BXY_1226300R	GCATTCACTTTGACATCCC	
31	BXY_1693800	BXY_1693800F	GACGGTCTTGACTACACTCTT	Amiloride-sensitive sodium channel
		BXY_1693800R	GTTCCTCTTTGAATCCTTTC	
32	BXY_0992600	BXY_0992600F	ACAGTATTCTACCTCGGCTTCA	Dual pore potassium ion channel
		BXY_0992600R	GACCACATTTCCCGTCCA	
33	BXY_1342400	BXY_1342400F	CCAACAATGGGAGGACAA	Neurotransmitter-gated ion channel
		BXY_1342400R	TGGAATGGATAGGCGATG	
34	BXY_1410500	BXY_1410500F	ACGCTACTCAAGTGATGGTC	Peroxidase
		BXY_1410500R	AGTAGCACCTGGTCGGATA	
35	BXY_0223700	BXY_0223700F	AACTGAGGATAATGGGTGAT	Succinate dehydrogenase [ubiquinone] flavoprotein subunit
		BXY_0223700R	CTGATTCCAACTTGAGTTCAT	
36	BXY_1209100	BXY_1209100F	GGTTTTGGAAGACTTTTGAG	Succinate dehydrogenase [ubiquinone] flavoprotein subunit
		BXY_1209100R	TTGACCTGGCTTGATGAT	
37	BXY_1476300	BXY_1476300F	ATTCAGCCGATTTTGTCC	Voltage-dependent calcium channel
		BXY_1476300R	TTATACGATTCGCTTACTTCTT	
38	Actin	BXactinF	CGCAAATACTCCGTCTGGATTGG	reference gene
		BXactinR	TTCGTCGTACTCTTGCTTGGAGA	

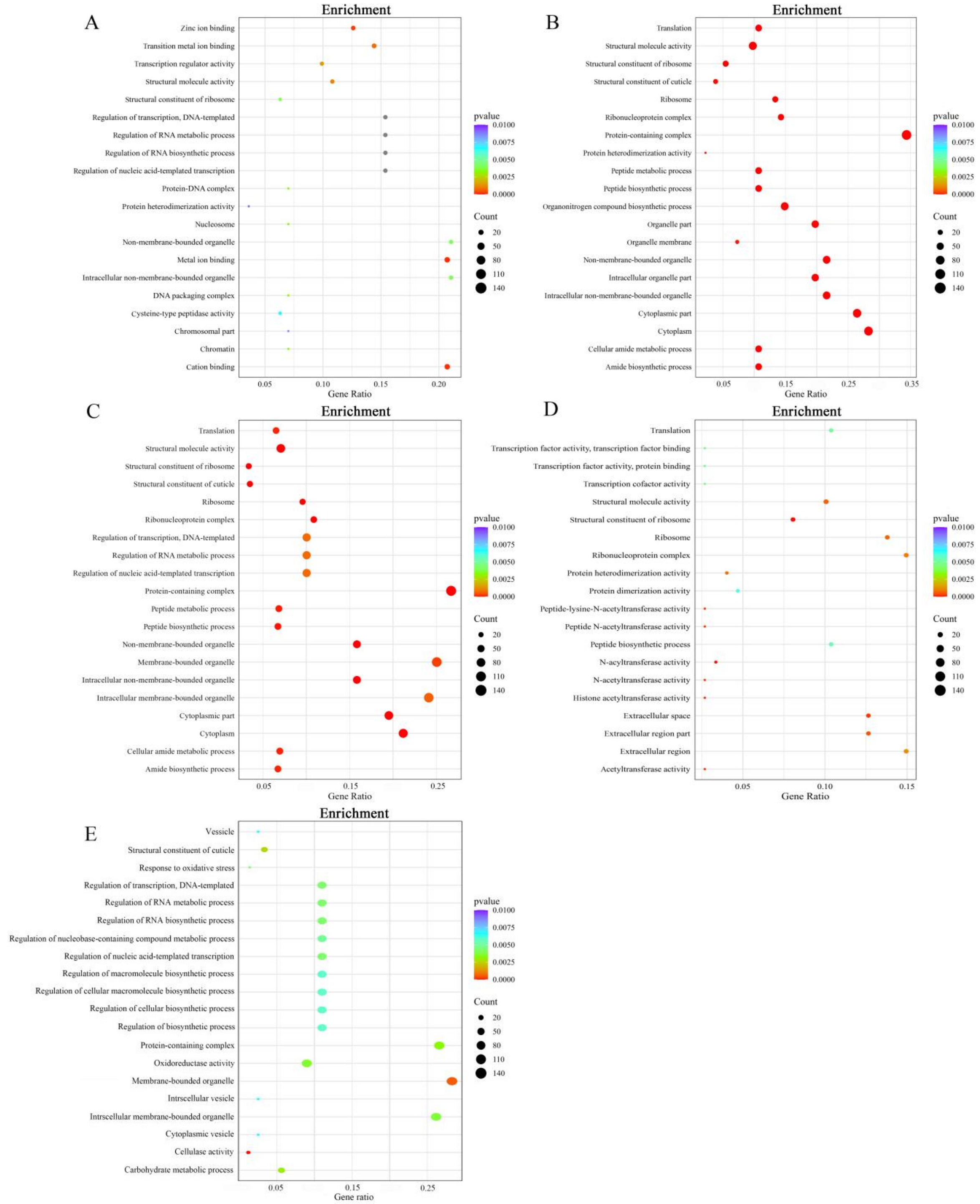

Figure S1. GO enrichment analysis of DEGs in *B. xylophilus* under different treatments (top 20). **(A)** SCF vs control; **(B)** SCA vs control; **(C)** SCY vs control; **(D)** SCF vs SCA; **(E)** SCF vs SCY.

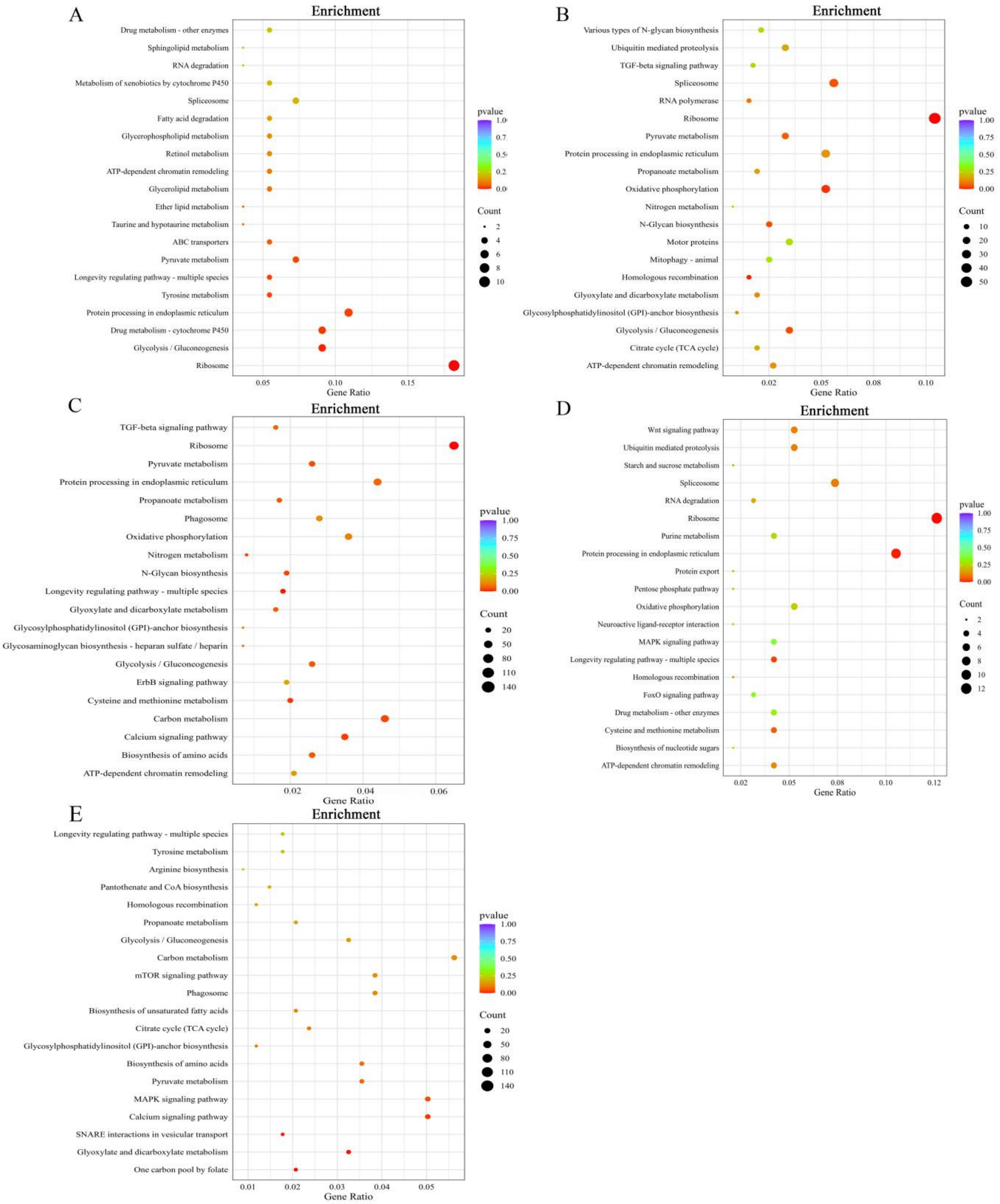

Figure S2. KEGG pathway enrichment analysis of DEGs in *B. xylophilus* under different treatments (top 20). **(A)** SCF vs control; **(B)** SCA vs control; **(C)** SCY vs control; **(D)** SCF vs SCA; **(E)** SCF vs SCY.
